# Statins Inhibit the Proliferation and Induce Cell Death of Human Papilloma Virus Positive and Negative Cervical Cancer Cells

**Published:** 2009-12

**Authors:** María Elena Crescencio, Emma Rodríguez, Araceli Páez, Felipe A. Masso, Luis F. Montaño, Rebeca López-Marure

**Affiliations:** 1 *Departamento de Biología Celular, Instituto Nacional de Cardiología “Ignacio Chávez”, México*; 2 *Laboratorio de Inmunobiología, Departamento de Biología Celular y Tisular, Facultad de Medicina, UNAM, México*

**Keywords:** statins, cell cycle, cell death, proliferation, cervical cancer, mevalonate pathway, oxidative stress, human papilloma virus

## Abstract

Statins, competitive inhibitors of 3-hydroxy-3-methylglutaryl-coenzyme A (HMG-CoA) reductase, have anti-tumoral effects on multiple cancer types; however, little is known about their effect on cervical cancer. We evaluated the effect on proliferation, cell cycle, oxidative stress and cell death of three statins on CaSki, HeLa (HPV^+^) and ViBo (HPV^−^) cervical cancer cell lines. Cell proliferation was assayed by crystal violet staining, cell cycle by flow cytometry and cell death by annexin-V staining. Reactive oxygen species (ROS) production was evaluated by the oxidation of 2,7-dichlorofluorescein diacetate and nitrite concentration (an indirect measure of nitric oxide (NO) production), by the Griess reaction. Inhibition of cell proliferation by atorvastatin, fluvastatin and simvastatin was dose-dependent. ViBo cells were the most responsive. Statins did not affect the cell cycle, instead they induced cell death. The antiproliferative effect in ViBo cells was completely inhibited with mevalonate, farnesyl pyrophosphate (FPP) and geranylgeranyl pyrophosphate (GGPP) treatments. In contrast, cell proliferation of CaSki and HeLa cells was partially (33%) rescued with these intermediates. The three statins increased ROS and nitrite production, mainly in the ViBo cell line. These results suggest that statins exert anti-tumoral effects on cervical cancer through inhibition of cell proliferation and induction of cell death and oxidative stress. Statins could be an aid in the treatment of cervical cancer, especially in HPV^−^ tumors.

## INTRODUCTION

Statins are 3-hydroxy-3-methylglutaryl-coenzime A reductase (HGM-CoA) inhibitors, the major rate-limiting enzyme that controls the conversion of HGM-CoA to mevalonic acid. Inhibition of mevalonate synthesis abolishes the production of isoprenoids farnesyl pyrophosphate (FPP) and geranygeranyl pyrophosphate (GGPP), crucial intermediates in cell signalization, differentiation and proliferation (1, 2).

Statins exert pleiotropic actions beyond its lipid-lowering effect. Several studies show that statins inhibit tumor cell proliferation (3–5); effect that depends on the type of cancer cells or the statin used (6–11). This antiproliferative effect has also been associated with inhibition of the metastasic potential and invasive properties of the tumor cells (12). Nevertheless, the mechanisms involved in these statin-induced effects remain unknown.

Mortality due to cervical cancer in mexican women has increased in the last two decades (13, 14), probably as a consequence of enhanced spreading of high-risk cancerogenic human papilloma virus genotypes. The aim of our work was to evaluate if statins with different chemical properties, could have an effect upon various cervical cancer cell lines. We evaluated the effect of atorvastatin, fluvastatin and simvastatin on the proliferation, cell cycle, oxidative stress and cell death on CaSki and HeLa cells that contain human papilloma virus (HPV) type, 16 and 18 respectively, and ViBo cells lacking HPV. All three statins inhibited the proliferation of the cervical cancer cell lines. ViBo cells were strongly inhibited by statins, as opposed to CaSki and HeLa cells which showed a lesser inhibition even though the differences between the two were not significant. The antiproliferative effect, induced by atorvastatin and simvastatin in CaSki and HeLa cells, was associated with lower cell death; whereas ViBo cells showed a higher cell death, but responded to the three different statins. In comparison to HeLa and ViBo cells statins induced a significant higher production of nitric oxide in CaSki cells. Interestingly, ViBo cells produced an increased amount of ROS that could be related to enhanced cell death. Intermediates of the mevalonate pathway fully rescued ViBo cell proliferation as opposed to HPV-containing tumor cells. These results indicate that statins could be useful in the treatment of cervical carcinomas, especially in HPV^−^ tumors.

## MATERIALS AND METHODS

Medium RPMI 1640 and trypsin were purchased from GIBCO/BRL (Grand Island, NY) and fetal bovine serum (FBS) from HyClone (Loga, Utah, USA). Annexin-V-Fluos was purchased from Roche (Mannheim, Germany). Atorvastatin, fluvastatin and simvastatin were purchased from Gödecke/Parke-Davis (Freiburg, Germany), Novartis (Barcelona, España) and MSD (Cramlington, UK), respectively. Sterile plastic material for tissue culture was from NUNC and COSTAR. Flow cytometry reagents and DNA reagent kit were purchased from Becton Dickinson, Immunocytometry Systems (San José, CA, USA). All other chemicals were purchased from Sigma Aldrich (St. Louis, MO, USA).

### Cell culture

CaSki and HeLa cells were purchased from American Type Culture Collection (ATCC, Rockville, MD). ViBo cells were established previously from a biopsy derived from a cervical tumor removed by surgery of a mexican patient diagnosed with epidermoid cervical carcinoma (15). All cervical cancer cells were cultured in RPMI-1640 medium supplemented with 5% of fetal bovine serum (FBS) and L-glutamine (2 mM). All experiments were performed in cultures plated at a cell density of 9 × 10^3^ cells/cm^2^.

### Cell proliferation

The number of cells was evaluated by crystal violet staining (16). Cells were plated in 96-multiwell plates and cultured with 0, 10, 20, 40, 80 and 160 μM of atorvastatin, fluvastatin and simvastatin for 24, 48 and 72 h. At the end of these treatments, cells were fixed with 100 μl of ice cold glutaraldehyde [1.1% in PBS (150 mM NaCl, 30 mM KCl, 15 mM Na_2_HPO_4_, 2 mM KH_2_PO_4_ pH 7.4)] for 15 min at 4°C. Plates were washed three times by submersion in de-ionized water, air-dried and stained for 20 min with 100 μl of a 0.1% crystal violet solution (in 200 mM phosphoric acid buffer at pH 6). After careful aspiration of the crystal violet solution the plates were extensively washed with de-ionized water, and air-dried prior to the solubilization of the bound dye with 100 μl of a 10% acetic acid solution incubated during 30 min. The optical density of the plates was measured at 595 nm in a multiplate spectrophotometer Bio-Tek Instruments, Inc. (Winooski, VT, USA). In the next experiments, cells were cultured with all statins at the optimal inhibitory concentration necessary to inhibit cell proliferation in 50% for 48 h (IC50).

### Analysis of cell cycle phases

DNA content was analyzed by propidium iodide (PI) staining followed by cytometric analysis using the Cycle TEST Plus (DNA reagent kit). After the treatment with statins for 48 h, cells were trypsinized and fixed with ethanol 70% in PBS for 30 min on ice. Cells were washed twice with PBS and incubated with RNAse (50 μg/ml) for 1 h at 37°C. Cells were then stained with PI (200 mg/l) for 2 min, washed twice with PBS and immediately subjected to cytometric analysis performed with a Becton Dickinson Facscalibur instrument (17).

### Determination of cell death

Translocation of phosphatidylserine on the membrane of cervical cancer cells (1.5 × 10^6^) was used to determine cell death (18). Cells were treated with statins at the optimal concentrations for 48 h before trypsinizing the cells; afterwards, these were washed with PBS and centrifuged at 200× g for 5 min. The cell pellet was resuspended in 100 μl of labeling solution [20 μl annexin-V-Fluos labeling reagent in 100 μl Hepes buffer (10 mM Hepes/NaOH, pH 7.4, 140 mM NaCl, 5 mM CaCl_2_), 1 μg/ml PI] and incubated for 15 min. The volume was increased to 500 μl with Hepes buffer and the samples were analyzed on a flow cytometer at 488 nm (excitation) and 515 nm bandpass filter for fluorescein detection, and a filter >560 nm for propidium iodide detection.

### Measurement of Reactive Oxygen Species

ROS generation was assessed using 2,7-dichlorofluorescein diacetate (DCFDA), a non-fluorescent probe, which upon oxidation by ROS and peroxides is converted to the highly fluorescent derivative DCF (19). Cells were incubated with DCFDA (10 μM) for 30 min at 37°C and washed twice with PBS. Cells were then cultured in the absence or presence of statins for 1 h. After an extensive wash fluorescence was evaluated in the Facscalibur. The mean intensity of the green fluorescence was determined using the Cell Quest software program and expressed as fluorescence channels (scale from 0 to 10,000 arbitrary units).

### Quantification of Nitrite

Cells were seeded in 96 well plates (NUNC) at a density of 1 × 10^5^ cells/well in RPMI without phenol red, 5% FBS, and with or without optimal concentrations of statins. After 24, 48 and 72 h, 100 μl of the conditioned medium were diluted 1:1 with 100 μl of Griess reactive and incubated for 15 min at room temperature. Previously, a standard curve was performed using known concentrations of sodium nitrite (range of 0.4 to 100 μM). The optical density of the plates was measured at 540 nm in a multiplate spectrophotometer. The absorbances of the concentrations of control and problem samples were plotted against the standard curve.

### Statistical analysis

All experiments were performed in triplicate in at least three independent trials. Some results are expressed as the average values ± standard deviation of the mean. Student’s T test was applied to determine statistical significance with p<0.01. ANOVA test was used when more than two groups were compared.

## RESULTS

### Statins inhibited the proliferation of cervical cancer cells

**Figure 1:**
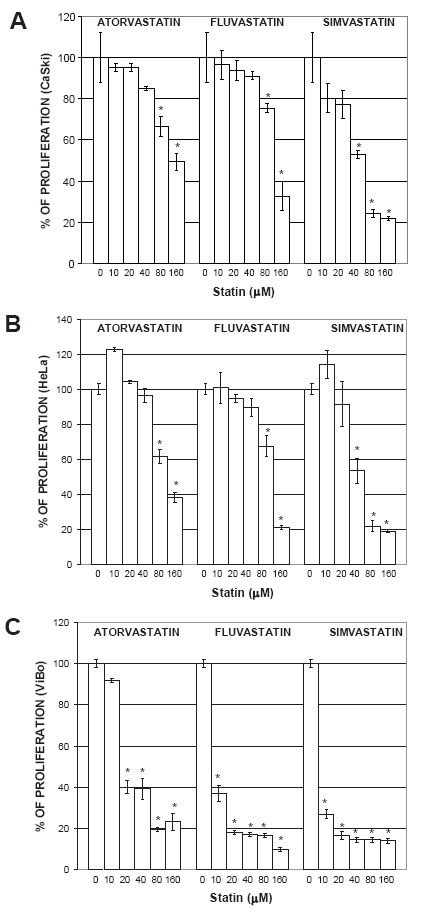
Effect of statins on cell proliferation. CaSki (A), HeLa (B) and ViBo cells (C) were treated with 0, 10, 20, 40, 80 and 160 μM atorvastatin, fluvastatin or simvastatin for 48 h. Cell proliferation was evaluated by crystal violet staining according to materials and methods. * Indicates a p<0.01 compared to control cells.

To determine the statin concentration needed to induce a 50% inhibition of proliferation (IC50), a dose-response curve was done with different statin concentrations (0 to 160 μM). Initial experiments showed that inhibition of proliferation started after 24 h. However, the stronger inhibitory effect was observed after 48 h (data not shown). In CaSki and HeLa cells, all statins inhibited the proliferation in a dose-dependent manner (Fig. 1A and 1B). In ViBo cells, which responded the most to the antiproliferative effect (Fig. 1C), an IC50 was reached with 6 μM simvastatin; greater concentrations were needed to reach the IC50 with the other statins (Table 1). Optimal IC50 statin concentrations were used in all the remaining experiments.

**Table 1:** IC50 Concentration of Statins

Concentration (μM)			
	Atorvastatin	Fluvastatin	Simvastatin

CaSki	154	114	41
HeLa	120	109	45
ViBo	29	8	6

Inhibitory concentration at which statins decreased the proliferation of cervical tumor cells by 50% (IC50). Cells were cultured with different concentrations of statins and proliferation was evaluated by crystal violet staining after 48 h. IC50 for each statin in all cell lines was calculated using the Biograph software version 2.5 (Bio-Tek Instruments, Inc). The results correspond to a representative experiment of three independent assays.

**Figure 2:**
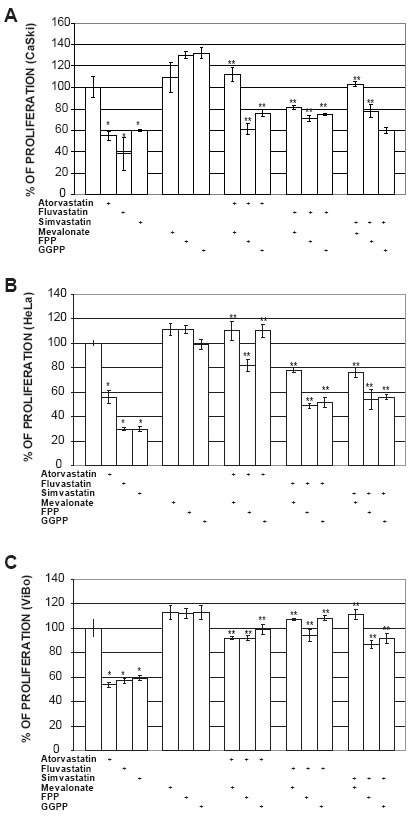
Effect of mevalonate pathway intermediates on the inhibition of proliferation induced by statins. Cells were cultured with mevalonate (100 μM), farnesyl pyrophosphate (FPP) (20 μM) or geranylgeranyl pyrophosphate (GGPP) (20 μM) 2 h before statin treatment and cell proliferation was evaluated by crystal violet staining after 48 h. A) CaSki cells, B) HeLa cells and C) ViBo cells. The results are expressed as percentage of proliferation with respect to untreated cells and are showed as mean ± SD of one experiment (n=5). The results correspond to a representative experiment of three independent assays. * Indicates a p<0.01 compared with control cells and ** a p<0.01 compared with statin-treated cells.

To determine if some intermediates of the mevalonate pathway could abrogate the antiproliferative effect induced by statins, cell cultures were treated with mevalonate, FPP or GGPP. The addition of mevalonate (100 μM), FPP (20 μM) or GGPP (20 μM) to statin-treated ViBo cells resulted in the complete recovery (100%) of proliferation (Fig. 2C). However, in fluvastatin and simvastatin treated CaSki and HeLa cells the intermediates abrogated the antiproliferative effect by 33% (Fig. 2A and 2B). This indicates that signaling pathway in HPV^+^ cells may be different from HPV^−^ tumor cells.

### Statins did not induce cell cycle arrest

**Figure 3:**
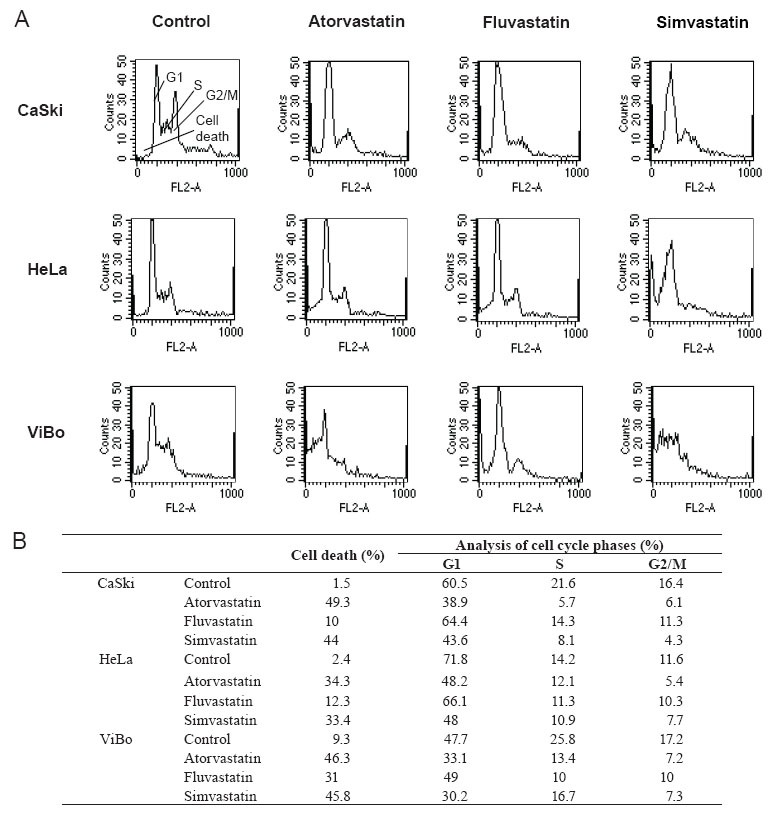
Effect of statins on the phases of the cell cycle. Cells were cultured without (Control) and with atorvastatin, fluvastatin or simvastatin and the percentage of cells in each phase of the cell cycle was evaluated by flow cytometry 48 h after. A) shows the histograms and B) shows the percentage of cells in each phase of the cell cycle obtained with the Modift software (Becton Dickinson). Upper left histogram shows the regions containing the cells in all the phases of the cell cycle (G1, S, G2/M) and cell death. The results correspond to a representative experiment of three independent assays.

To determine if cell proliferation inhibition is associated with cell cycle interference, cervical cancer cells were treated with statins and the percentage of cells in the different phases of the cycle was evaluated. The percentage of cells in the G1 and S phases decreased in all treated cell lines in comparison to control cells (Fig. 3). This decrease was not associated with an increase in the percentage of cells in G2/M phases of the cell cycle, but was related to an increase in the percentage of cell death. The toxic effect of fluvastatin was considerably lower than the other two statins. It was interesting to observe that the percentage of cell death in HPV^+^ cell lines was in the 10–12% range as opposed to 31% reached in the HPV^−^ cell line (Fig. 3B). The cell death, induced by atorvastatin and simvastatin, was in the 33–49% range.

### Statins induced cell death

**Figure 4:**
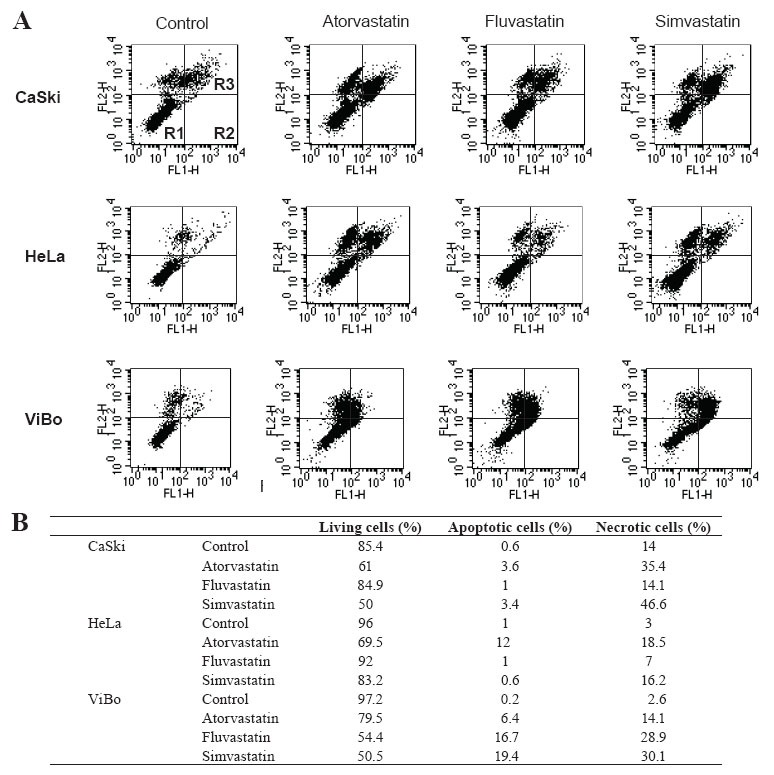
Effect of statins on cell death evaluated by annexin-V-fluos. Cells were cultured without (Control) and with statins for 48 h. A) Cells were stained with annexin-V-fluos (FL1-H) and propidium iodide (PI) (FL2-H). Lower left panel shows living cells (R1), lower right panel, apoptotic cells (R2), and upper right panel, necrotic cells (R3). B) Graphic representation of the percentage of cells in each panel obtained by flow cytometry with the Modift software. The results correspond to a representative experiment of three independent assays.

To determine if the toxicity induced by statins was due to apoptotic or necrotic cell death, statin-treated tumoral cells were stained with annexin-V-fluos and propidium iodide. Atorvastatin and simvastatin decreased by approximately 20–25% the percentage of living cells in all cell lines. Fluvastatin did not have an effect on HPV^+^ cells, but in ViBo cells it diminished viability by 42% (p< 0.01). This decrease in cell viability was associated with an increase in apoptotic and necrotic cell death (Fig. 4). It was interesting to observe that increased necrotic cell death was stronger than apoptotic cell death in the three cell lines.

### Statins induced an increase of oxidative stress

**Figure 5:**
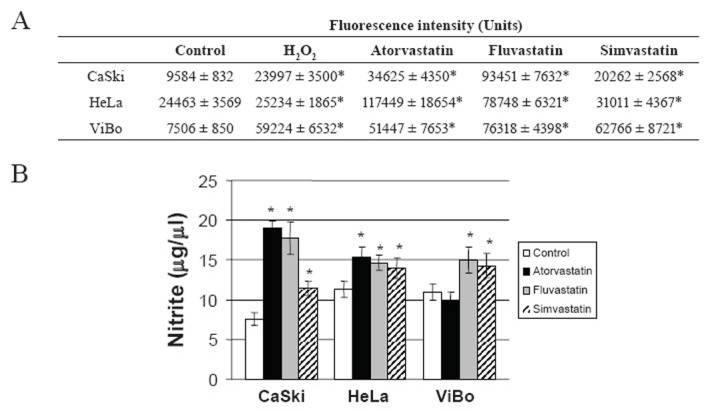
ROS and nitrite production by statins-treated cell lines. A) ROS production was evaluated using DCFDA and flow cytometry. The results are expressed as fluorescence intensity and are showed as mean ± SD of one experiment (n=5). Fluorescence intensity (units) was calculated by multiplying the number of events by the mean fluorescence intensity value in a region selected manually with positive fluorescence in the channel FL-1 (DCF). The results correspond to a representative experiment of three independent assays. Cells without DCFDA were used as a negative control. B) Nitrite concentration was determined by Griess reagent reactivity (see methods). The results are expressed as mean ± SD of one experiment (n=5). We show a representative experiment of three independent assays. *Indicates a p < 0.01 in comparison with the control (cells without treatment).

To determine if cell death induced by statins was a consequence of oxidative stress, ROS production and nitrite concentration were evaluated. All statins increased ROS (Fig. 5A) and nitrite production in the cell lines (Fig. 5B). The increase in NO production was considerably higher in CaSki cells than in HeLa and ViBo cells. Interestingly, ROS production was significantly higher in HPV^−^ ViBo cells (p<0.01) in comparison to HPV^+^ cells.

## DISCUSSION

Statins, fundamental in hypercholesterolemia treatment, also inhibit the proliferation of various tumor cell lines by inducing cell cycle arrest and by triggering apoptosis (20). Statins inhibit the formation of mevalonate, a precursor of cholesterol and non-steroidal isoprenoid, compounds that are required for cell proliferation. Mevalonate depletion results in a G1 phase cell cycle arrest which is mediated, on one hand, by impaired activity of cyclin-dependent kinase 2 (CDK2), and on the other, by decreased expression of positive regulators of G1 to S phase progression (21). We found that atorvastatin, fluvastatin and simvastatin inhibited the proliferation of three cervical cancer cell lines in a dose-dependent manner. A similar antiproliferative effect has been observed with statins in other carcinomas such as embryonal carcinoma (NTERA-2), colorectal cancer (HT-29), breast cancer (MCF-7), prostate cancer (LNCaP) and glioma cells (22–25). Our results showed that statins decreased the percentage of cells in the G1 and S phases of the cell cycle, indicating that statins are able to inhibit the transition to S phase and therefore, the synthesis of DNA, which can be related with the inhibition of cell proliferation and cell death.

HPV negative ViBo cells responded the most to statin induced antiproliferative and cytotoxic effects in comparison to CaSki and HeLa cells infected with HPV types 16 and 18, respectively. Probably the absence of papilloma virus in tumor cells favors an enhanced response to antiproliferative molecules such as statins. We believe our HPV^+^ tumoral cell lines have alterations in the proteins regulating the cell cycle thus responding differently to the inhibition effect of statins. The expression of E6/E7 proteins decreases p53 and Rb protein expression, respectively (26, 27); both are important tumor suppressor proteins. It is probable that the expression of p53 and Rb proteins in ViBo cells it is normal, and therefore, they have a better response to antiproliferative factors.

Our results showed that simvastatin was the most effective inhibitor of cervical tumor cell proliferation. Simvastatin, a hydrophobic statin, is a type I inhibitor, whereas atorvastatin and fluvastatin are type 2 inhibitors (28). There are important biochemical differences between the two types, which could modify their uptake mechanisms (29). For example, it has been shown that pravastatin is less able to enter the cells compared with lovastatin, mevastatin, or simvastatin, possibly because it is highly hydrophilic (30, 31).

The reversal of the antiproliferative effect induced by statins with mevalonate in cervical cancer cells, indicates that mevalonate metabolites are required for cellular survival in these cells. In contrast, the antiproliferative effect induced by statins was not completely abrogated by FPP and GGPP, suggesting that other metabolites could have an important role in the inhibition induced by statins in these cell lines. In the specific case of lovastatin-induced cytotoxicity, addition of squalene or cholesterol had no reverting effect, thus suggesting that the inhibition of cholesterol production is not critical (32). In other cancer cells, both FPP and GGPP rescues cell proliferation, indicating that these metabolites participate directly in the inhibition of the proliferation induced by statins (33). It has been demonstrated that the replenishment of the intracellular pool of GGPP, which is depleted by statins, might be highly relevant for the control of the apoptotic mechanism (34–36). Our results showed that ViBo cells, which are HPV^−^, were the only ones with a 100% antiproliferative effect reversal induced by statins, when treated with the above mentioned metabolites. So far, and to our knowledge, there is no explanation on the effect of the HPV virus upon the mevalonate pathway.

Besides the cytostatic effect induced by the three statins on cervical cancer cells, we also observed a cytotoxic effect. Interestingly the three statins did not induce an arrest in any phase of the cell cycle, but induced apoptotic and necrotic cell death. It has been shown that statins induce apoptotic cell death in different cell lines (8–10). Dimitroulakos *et al* observed that lovastatin induces cytotoxicity on the SIHA and HT1 cervical cancer cell lines (5). Similarly, statins also induce apoptosis of clonal B lymphocytes in patients with chronic lymphocytic leukemia (37). However, a study performed with different myeloma cell lines treated with simvastatin, showed that some had apoptotic death whereas others displayed a more necrotic form of death (8); these results match ours. In MCF-7 breast cancer cells, statins inhibited proliferation associated to an increase in caspase-3-like activity, DNA fragmentation, and apoptotic and necrotic cell death (38). Simvastatin strengthens TNF-alpha-induced apoptosis through the down-regulation of NF-κB-dependent antiapoptotic gene products (39), suggesting a role in the prevention and treatment of cancer cells through NF-κB modulation (39). A similar observation with natural statins (simvastatin, mevastatin, lovastatin and pravastatin), but not synthetic statins (fluvastatin and atorvastatin), has been reported (40). Our results suggest that in cervical carcinoma, statins have a differential effect depending on their concentration, group and class, and most importantly, the presence or not of HPV.

It has been shown that ROS and other oxidants can cause oxidation of lipids, proteins and DNA with following tissue damage. Toxic products of oxidation proceed cytostatic effects causing membrane damage and lead into cell death via apoptosis or necrosis (41).

The treatment of cervical cells with all the statins induced an increase of the oxidative stress mediated by an increase of ROS and NO production. A recent report has shown that lovastatin-induced apoptosis is due to intracellular ROS increase and that these effects that can be associated to the death observed in k-ras-transformed thyroid cells (42). In other work, we also showed that statins can induce oxidative stress associated with death in MCF-7 cells (23). These results indicate that the toxic effect induced by statins is mediated by an oxidative stress in cervical cancer cells.

## CONCLUSION

Atorvastatin, fluvastatin and simvastatin, were effective inhibitors of the *in vitro* proliferation of cervical cancer cells. They were cytotoxic, and favored necrotic cell death. Their efficacy depended on the presence or not of HPV. Oral administration of statins, either alone or combined with antineoplasic agents, could be a novel, safe and effective promising approach to cervical cancer treatment.
